# Bioequivalance and pharmacokinetic study of febuxostat in human plasma by using LC-MS/MS with liquid liquid extraction method

**DOI:** 10.1186/2193-1801-2-194

**Published:** 2013-04-30

**Authors:** Babu Rao Chandu, Kanchanamala Kanala, Nagiat T Hwisa, Prakash Katakam, Mukkanti Khagga

**Affiliations:** Faculty of Pharmacy, University of Al-Zawia, Zawiya, Libya; Jawaharlal Nehru Technological University Anantapur, Andhrapradesh, 515002 India; Ratnam Institute of Pharmacy, Pidatapolur, Muthukur, Nellore, Andhrapradesh 524346 India; Jawaharlal Nehru Technological University Hyderabad, Andhrapradesh, 500072 India

**Keywords:** LC-MS/MS, Febuxostat, Human plasma, Bioequivalance, Pharmacokinetics

## Abstract

A bioequivalence study was proved of generic Febuxostat 80 mg tablets (T) in healthy volunteers.For this purpose, Authors developed a simple, sensitive, selective, rapid, rugged and reproducible liquid chromatography–tandem mass spectrometry method for the quantification of Febuxostat (FB) in human plasma using Febuxostat D7 (FBD7) as an internal standard (IS) was used. Chromatographic separation was performed on Ascentis Express C18 (50x4.6 mm, 3.5 μ) column. Mobile phase composed of 10 mM Ammonium formate: Acetonitrile (20:80 v/v), with 0.8 mL/min flow-rate. Drug and IS were extracted by Liquid- liquid extraction. FB and FBD7 were detected with proton adducts at m/z 317.1→261.1 and 324.2→262.1 in multiple reaction monitoring (MRM) positive mode respectively. The method was validated with the correlation coefficients of (r^2^) ≥ 0.9850 over a linear concentration range of 1.00-8000.00 ng/mL. This method demonstrated intra and inter-day precision within 2.64 to 3.88 and 2.76 to 8.44% and accuracy within 97.33 to 99.05 and 100.30 to 103.19% for FB. This method is successfully applied in the Bioequivalence study of 9 human volunteers.

## Introduction

**Figure 1:**
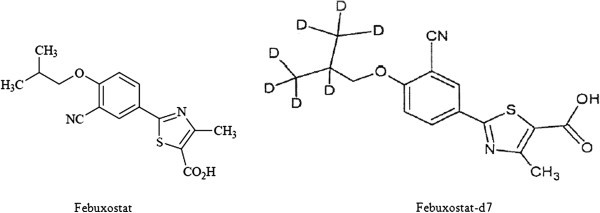
**Chemical structures of Febuxostat and Febuxostat-d7.**

Febuxostat is chemically 2-(3-cyano-4-isobutoxyphenyl)-4-methyl-1,3- thiazole-5- carboxylic acid. It is available in the form non hygroscopic crystalline nature and freely soluble in dimethylformamide, soluble in dimethylsulfide, sparingly soluble in ethanol, slightly soluble in methanol and acetonitrile, and practically insoluble in water (Tayar et al. 2012;Hussar & Bilbow 2009;Tomillero & Moral 2009). Febuxostat is orally administered a non-purine, selective inhibitor of xanthine oxidase being developed for the management of hyperuricaemia in patients with gout (Zhu et al. 2009;Hoshide et al. 2004;Becker et al. 2005;Grabowski BA, et al. et al. 2011;Kamatani N, et al. et al. 2011;). The molecular formula is C_16_H_16_N_2_O_3_S (Figure 1). Febuxostat, is available in two dosage strengths; 40 mg and 80 mg. The absorption of radiolabeled Febuxostat following oral dose administration was estimated to be at least 49% (based on total radioactivity recovered in urine). Maximum plasma concentrations of Febuxostat occurred between 1 to 1.5 hours post-dose. After multiple oral 40 mg and 80 mg once daily doses, Cmax is approximately 1.6 ± 0.6 mcg per mL (N = 30), and 2.6 ± 1.7 mcg per mL (N = 227), respectively. Absolute bioavailability of the Febuxostat tablet has not been studied. Following multiple 80 mg once daily doses with a high fat meal, there was a 49% decrease in Cmax and an 18% decrease in AUC, respectively. However, no clinically significant change in the percent decrease in serum uric acid concentration was observed (58% fed vs. 51% fasting). The mean apparent steady state volume of distribution (V_SS_/F) of Febuxostat was approximately 50 L (CV ~40%). The plasma protein binding of Febuxostat is approximately 99.2%, (primarily to albumin), and is constant over the concentration range achieved with 40 mg and 80 mg doses. Febuxostat is extensively metabolized by liverand eliminated by both hepatic and renal pathways. The apparent mean terminal elimination half-life (t_1/2_) of Febuxostat was approximately 5 to 8 hours (Khosravan R et al. 2012;).

Literature survey reveals that, there are few methods were reported for quantification of Febuxostat by using UV (Bagga et al. 2011;Sheth et al. 2012), Liquid chromatography (LC) (Gunda et al. 2012a;Kumaraswamy Gandla et al. 2012;Cong et al. 2010;Naresh Chandra Reddy & Chandra Sekhar 2012;Nageswara Rao et al. 2012;Gunda et al. 2012b;Muvvala1 et al. 2012;Yamamoto et al. 1995;Menon et al. 2011;Mathrusri Annapurna et al. 2012), UPLC (Sahu et al. 2012), UPLC-MS (Lukram et al. 2012;Zhang et al. 2012). Liquid chromatography -Mass spectrometry (LC) (Kadivar et al. 2011;Ding et al. 2012;Wang et al. 2012). These methods have been reported for the quantitative estimation of Febuxostat in pharmaceutical (Bagga et al. 2011;Sheth et al. 2012;Gunda et al. 2012a;Kumaraswamy Gandla et al. 2012;Cong et al. 2010;Naresh Chandra Reddy & Chandra Sekhar 2012;Nageswara Rao et al. 2012;Gunda et al. 2012b;Muvvala1 et al. 2012;Sahu et al. 2012;Kadivar et al. 2011) and biological fluids (Yamamoto et al. 1995;2011;Menon et al. Lukram et al. 2012;Zhang et al. 2012;Ding et al. 2012;Wang et al. 2012). Among all Quantification of Febuxostat in biological matrices by using LC-MS/MS (Ding et al. 2012;Wang et al. 2012) were reported. Xitao ding et.al (Ding et al. 2012) developed the method in rat plasma using protein precipitation extraction method by using acetonitrile as an precipitating agent. They developed with the linearity range of 10–2000 ng/mL for Febuxostat and used midazolam as internal standard and applied to determinate of Febuxostat in rat plasma for pharmacokinetic study. Wang H et.al. (2012) developed the method in human plasma using protein precipitation extraction method by using acetonitrile as an precipitating agent. They developed the method with linear range of 10.00-5000.00 ng/mL for Febuxostat and used Febuxostat-d7 as an internal standard and performed the pharmacokinetic study in healthy chinese volunteers.

However LC-MS/MS has gained importance for the quantitative estimation of drugs in various biological matrices including plasma, serum, urine, and ocular fluids, due to its high sensitivity, selectivity and reproducibility. The published methods (Ding et al. 2012;Wang et al. 2012) are precipitation methods by using LC-MS. Precipitation method has some disadvantages rather than Liquid-liquid extraction in-terms of extraction efficacy, matrix effect. The aim of the proposed method is extraction of Febuxostat in human plasma by Liquid-liquid extraction method and used internal standard as deuterated compound Febuxostat–d7. The proposed method is most simple, highly sensitive, wide linear and high selective extraction method with good recovery, fully validated as per FDA guidelines and was successfully applied in Bio-equivalence study of 80 mg formulations in healthy human volunteers.

## Materials and methods

### Chemicals and reagents

Febuxostat obtained from AMI Life sciences Pvt.Lt.D Baroda,Gujarat India and Febuxostat D7 Obtained from Euroasian Chemicals Pvt.Ltd, Mumbai , India. HPLC grade methanol, Acetonitrile were purchased from Jt. Baker Mallinckrodt Baker, Inc. Phillipsburg, NJ, USA. Ammonium formate, Formic acid was purchased from S.D fine chemicals Mumbai. Methyl t-butyl ether was purchased from Merck speciality private limited, worli, Mumbai. Ultra pure water from Milli-Q system (Millipore, Bedford, MA, USA) was used through the study. All other chemicals in this study were of analytical grade.Human plasma was obtained from Doctors pathological lab , Hyderabad.

### Drugs

The reference Urolac® immediate release tablet formulation was manufactured by Takeda Pharmaceuticals, America, INC (USA), (batch number R1785, manufacture date Jun 07, 2011, and expiry date Jun 07, 2013). The test formulation was manufactured byAPL Research center. Each immediate release tablet of both formulations contained Febuxostat equivalent to 80 mg. The clinical study was conducted at Clinical and Pharmacological Research Unit, Acron Accunova, Manipal.

### Instrumentation

HPLC system (1200 Series Agilent Technologies, Germany) connected with triple quadrupole mass spectrometer instrument (API 4000, Toronto, Canada). Data processing was performed with the Analyst 1.4.1 software package (SCIEX). Ionization was performed by Electro spray positive mode with Unit Resolution.

### Detection

Mass parameters were optimized to get the product ions of m/z: 261.1, m/z: 262.1 from its respective precursor ions of FB [M + H]^+^ (m/z: 317.1) and FBD7 [M + H]^+^ (m/z: 324.2) with Source temperature 500°C, Ion Spray voltage 5500 volts, Heater gas, Nebulizer gas 30 psi each, Curtain gas 20 psi, CAD gas 6 psi, (all gas channels with nitrogen) Source flow rate 500 μL/min without split, Entrance potential 10 V, Declustering potential 45 V for analyte and 55 V for internal standard, Collision energy 28 V for both analyte and internal standard, Collision cell exit potential 12 V for analyte and 14 V for internal standard.

### Chromatographic conditions

Chromatography was performed on Ascentis Express C18 (50x4.6 mm, 3.5 μ) analytical column at 40°C, with 10 mM Ammonium formate: Acetonitrile (20:80 v/v) as mobile phase at a flow rate of 0.8 mL/min. FBD7 was used as an internal standard in terms of chromatography and extractability. The drug and internal standard was eluted at 0.6 ± 0.2 min with 2.5 min total run time.

### Preparation of standards and quality control (QC) samples

Standard stock solutions of FB (1000.00 μg/mL) and FBD7 (100.00 μg/mL) was prepared in methanol. The internal standard spiking solution (1000.00 ng/mL) was prepared in 50% acetonitrile from FBD7 standard stock solution (100.00 μg/mL). Standard stock solutions and Internal standard spiking solutions were stored in refrigerator conditions (2–8°C) until analysis. Standard stock solution of FB was added to screened drug-free human plasma to obtain concentration levels of 1.00, 2.00, 20.00, 100.00, 200.00, 400.00, 1000.00, 3000.00, 6000.00, and 8000.00 ng/mL for analytical standards and 1.00, 3.00, 4000.00, 4800.00 ng/mL for Quality control standards and stored in a −30°C freezer until analysis. Respective aqueous standards were prepared in reconstitution solution (10 mM ammonium formate: acetonitrile (20:80) and stored in refrigerator conditions 2–8°C until analysis.

### Sample preparation

Liquid-liquid extraction was used to isolate drug and IS from human plasma. For this purpose, 100 μL of IS (1000.00 ng/mL) and 100 μL of plasma sample (respective concentration) was added into labeled polypropylene tubes and vortexed briefly. Followed by, 100 μL of 0.1% formic acid, 2.0 mL of extraction solvent (methyl tertiary butyle ether) were added and vortexed for 10 min. Then the samples were centrifuged at 4000 rpm for 5 min at 20°C temperature. Subsequently, the supernatant from each sample was transferred into respective polypropylene tubes. After that, all the samples were kept for evaporation under nitrogen at 40°C. The dried residue was reconstituted with 1000 μL of reconstitution solution and vortexed briefly. Finally, the extracted sample was transferred into auto sampler vials and injected into LC-MS/MS.

### Selectivity and specificity

The selectivity of the method was determined by six different human blank plasma samples, which were pretreated and analyzed to test the potential interferences of endogenous compounds co-eluting with analyte and IS. Chromatographic peaks of analyte and IS were identified based on their retention times and MRM responses. The peak area of FB at the respective retention time in blank samples should not be more than 20% of the mean peak area of LOQ of FB. Similarly, the peak area of FBD7 at the respective retention time in blank samples should not be more than 5% of the mean peak area of LOQ of FBD7.

### Recovery

The extraction recovery of FB and FBD7 from human plasma was determined by analyzing quality control samples. Recovery at three concentrations (30.00, 4000.00, and 4800.00 ng/mL) was determined by comparing peak areas obtained from the plasma sample and the standard solution spiked with the blank plasma residue. A recovery of more than 85% was considered adequate to obtain required recovery.

### Limit of detection (LOD) and limit of quantification (LOQ)

The limit of detection (LOD) is a parameter that provides the lowest concentration in a sample that can be detected from background noise but not quantitated. LOD was determined using the signal-to-noise ratio (s/n) of 3:1 by comparing test results from samples with known concentrations of analytes with blank samples.

The limit of quantitation (LOQ) is defined as the lowest concentration of analyte that can be determined with acceptable precision and accuracy. The LOQ was found by analyzing a set of mobile phase and plasma standards with a known concentration of FB.

### Matrix effect

To predict the variability of matrix effects in samples from individual subjects, matrix effect was quantified by determining the matrix factor, which was calculated as follows:

Six lots of blank biological matrices were extracted each in triplicates and post spiked with the aqueous standard at the Low,High QC level, and compared with aqueous standards of same concentration. The overall precision of the matrix factor is expressed as coefficient of variation (CV %) and %CV should be < 15%.

### Calibration curve, precission and accuracy

The calibration curve was constructed using values ranging from 1.00 to 8000.00 ng/mL of FB in human plasma. Calibration curve was obtained by linear model with weighted 1/x^2^ regression analysis. The ratio of FB / FBD7 peak area was plotted against the ratio of FB concentration in ng/mL. Calibration curve standard samples and quality control samples were prepared in replicates (n = 6) for analysis. Precision and Accuracy for the back calculated concentrations of the calibration points, should be within ≤15 and ± 15% of their nominal values. However, for LLOQ, the Precision and Accuracy should be within ≤ 20 and ± 20%.

### Stability (Freeze - thaw, Auto sampler, Bench top, Long term) of FB in plasma

Low quality control and high quality control samples (n = 6) were retrieved from a deep freezer after three freeze-thaw cycles according to the clinical protocol. Samples were stored at −30°C in three cycles of 24, 36 and 48 hr. In addition, the long-term stability of FB in quality control samples was also evaluated by analysis after 55 days of storage at −30°C. Autosampler stability was studied following 71.5 hr storage period in the autosampler tray with control concentrations. Bench top stability was studied for 24.5 hr period with control concentrations. Stability samples were processed and extracted along with the freshly spiked calibration curve standards. The Precision and Accuracy for the stability samples must be ≤ 15 and ± 15% respectively of their nominal concentrations.

### Study subjects

The study was carried out in accordance with the current revision of the Declaration of Helsinki concerning medical research in humans. Study protocol was approved by IEC (Institutional Ethics committee) as per DCGI (Drug control general of india). Fourteen healthy male subjects, were included in the pilot study. All volunteers gave a written informed consent prior to participation, after they had been informed of the nature and details of the study which they thoroughly understood. Subject screening examinations were performed by a study. Clinician at acron accunova manipal center. All clinical laboratory tests were performed by the ISO 15189 certified laboratories, Department of Pathology, acron accunova manipal Bangalore. The daily results of the clinical laboratory tests including the quality control data were verified by its own independent quality assurance personnel before reporting.

Subject inclusion criteria included Indian male, aged between 18–45 years, no consumption of drugs or food supplements for 4 weeks prior to the study, and no participation in any bioavailability or bioequivalence study at least 30 days prior to the present study.

The exclusion criteria included history of hypersensitivity to Febuxostat and/or related chemical structure and/or any of the components of the product, history or concurrent symptoms of cardiovascular, liver, kidney, gastrointestinal or hematological disorders and/or any disease that might affect the bioavailability of drug, subjects with malignancy, AIDs, allergy, vital sign abnormalities, or clinically significant abnormal values during pre-study screening, smoker (>10 cigarettes/day) or smoker of < 10 cigarettes/day who could not quit at least 7 days before study and throughout study (including washout period), regular alcohol consumption (more than 1 time/week) or alcohol consumption within 7 days prior to the study, coffee consumption within past 7 days, and drug addiction.

### Study design

The study was conducted as an open label, randomized two-period, two-sequence, single-dose crossover bioequivalence study under fasting condition, and a wash-out period of 7 days. All subjects arrived at the clinical research laboratory, at least 12 h prior to the start of the study. They were housed in an air-conditioned facility and were given a standard dinner, which was finished at least 10 h before dosing in each period of the study. On the day of drug dosing in period 1, volunteers were randomly assigned to one of two treatment sequences (TR (sequence 1) or RT (sequence 2)), as indicated in a pre-printed randomization scheme, which was generated using block randomization with the blocks of size 4 and 6, and the allocation ratio of 1:1. Subjects in sequence 1 received treatment T at the first dosing period and then crossed over to receive treatment R at the second dosing period (after the 7-day washout period). Subjects in sequence 2 received treatments in the order of R and T at the two dosing periods. The subjects were administered the assigned Febuxostat formulation with 240 mL of plain drinking water. After the intake of the study formulations, the oral cavity was checked to ensure completion of the administration process. Subjects were required to refrain from lying down during the first 4 h after dosing.

No meal was permitted until 4 h after dosing. Drinking water was restricted from 1 h before dosing till 2 h after dosing and *ad libitum* thereafter. Excess water intake (> 100 mL/h) was not permitted. Lunch, snacks, and dinner were served as per the scheduled time. All subjects abstained from any xanthine-containing food or beverages for at least 72 h and alcoholic products for at least 7 days prior to formulation administration and throughout the sampling schedule during each period. They were informed not to take any drug at least 30 days prior to the study, especially phenobarbital, rifampicin or gemfibrozil. Subjects abstained from the use of tobacco- or nicotine-containing products for 7 days prior to dosing and during confinement in the clinical research laboratory. No concomitant medication was permitted during the study period.

### Blood sampling

Blood samples were collected as the pre-dose (0) hr 5 minutes prior to dosing followed by further samples at 0.167, 0.25, 0.333, 0.5, 0.75, 1, 1.33, 1.667, 2, 2.5, 3, 4, 5, 6, 8, 10, 12, 14, 18, 24, 30 and 36.0 hours. After dosing 2.5 mL blood was collected each time in vaccutainers containing K_2_EDTA. A total of 44 (23 time points for Reference, 23 time points for Test) time points were collected by using centrifugation 3200 rpm, 10°C, 10 min and stored below −30°C until sample analysis.Test and Reference Febuxostat tablets were administered to same human volunteers under fasting conditions separately with proper washing periods as per protocol (Comparative, Randomized, 2-way crossover) approved by IEC. During the sample collection, all subjects were under medical supervision. Vital signs were examined at scheduled time as described in the protocol.

#### Tolerability assessments

Throughout the study, subjects were monitored by a clinician, a clinical pharmacist, and 4 nurses. Tolerability was determined by monitoring of vital signs (sitting blood pressure, heart rate, and axillary body temperature), and physical examinations at baseline and at the end of each study period. Subject interviews were also conducted regarding the potential occurrence of adverse events (AEs) at each Febuxostat study period. *Serious AEs* (SAEs) were considered to be when the subject outcome was death, life threatening, requiring hospitalization, leading to disability, or requiring medical intervention to prevent permanent impairment or damage. All AEs and SAEs were recorded in the source data record and on the case-report form, and their relationship to the study drug was determined by the study physician who was blinded to the randomization schedule.

### Pharmacokinetics and statistical analysis

Pharmacokinetics parameters from the human plasma samples were calculated by a non-compartmental statistic model using WinNon-Lin5.0. software (Pharsight, USA). Blood samples were taken for a period of 3 to 5 times the terminal elimination half-life (t_1/2_) and it was considered as the area under the concentration time curve (AUC) ratio higher than 80% as per FDA guidelines. Plasma FB concentration-time profiles were visually inspected, and C_max_ and T_max_ values were determined. The AUC_0–t_ was obtained by the trapezoidal method. AUC_0–∞_ was calculated up to the last measureable concentration and extrapolations were obtained using the last measureable concentration and the terminal elimination rate constant (K_e_) it was estimated from the slope of the terminal exponential phase of the plasma of the FB concentration-time curve (by means of the linear regression method). The terminal elimination half-life (t_1/2)_, was then calculated as 0.693/K_e_. Regarding AUC_0–t,_ AUC_0–∞_ and C_max_ bioequivalence were assessed by means of analysis of variance (ANOVA) and calculating the standard 90% confidence intervals (90% CIs) of the ratio's test/reference (logarithmically transformed data). The bioequivalence was considered when the ratio of averages of log transformed data was within 80-125% for AUC_0–t,_ AUC_0–∞_ and C_max_ (Guidance for industry 2002;Guidance for industry 2003).

## Results and discussion

### Method development

**Figure 2:**
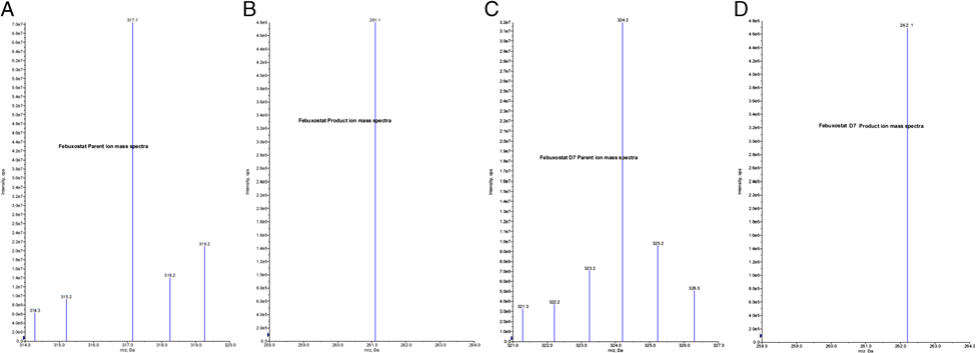
**Mass spectra of (A) Febuxostat parent ion, (B) Febuxostat product ion, (C) Febuxostat-d7 parent ion and (D) Febuxostat-d7 product ion.**

LC-MS/MS has been used as one of the most powerful analytical tools in clinical pharmacokinetics for its selectivity, sensitivity and reproducibility. The goal of this work is to develop and validate a simple, sensitive, rapid, rugged and reproducible assay method for the quantitative determination of FB from human plasma samples. Chromatographic conditions, especially the composition and nature of the mobile phase, usage of different columns, different extraction methods such as solid phase, Precipitation, Liquid-liquid extraction methods were optimized through several trials to achieve the best resolution and increase the signal of FB and FBD7. The MS optimization was performed by direct infusion of solutions of both FB and FBD7 into the ESI source of the mass spectrometer. Product ion spectrum for FB and FBD7 yielded high-abundance fragment ions of *m*/*z* 261.1 and *m*/*z* 262.1 respectively (Figure 2A,D). After mass spectrometer parameters optimized, chromatographic conditions such as mobile phase optimization, column optimization, extraction method optimization was performed to obtain a fast and selective LC method. A good separation and elution were achieved using 10 mM Ammonium formate: Acetonitrile (20:80 v/v) as the mobile phase, at a flow-rate of 0.8 mL/min and injection volume of 5 μL. Ascentis Express C18 (50x4.6 mm, 3.5 μ) column and Liquid liquid extraction method was optimized for the best chromatography.

### Method validation

The developed method was validated over a linear concentration range 1.00-8000.00 ng/mL. The validation parameters include Selectivity and Specificity, Limit of Detection (LOD) and Quantification (LOQ), Matrix effect, Precision and Accuracy, Recovery, Stability (Freeze - thaw, Auto sampler, Bench top, Long term) was evaluated under validation section (Guidance for industry 2001).

### Selectivity and specificity

**Figure 3:**
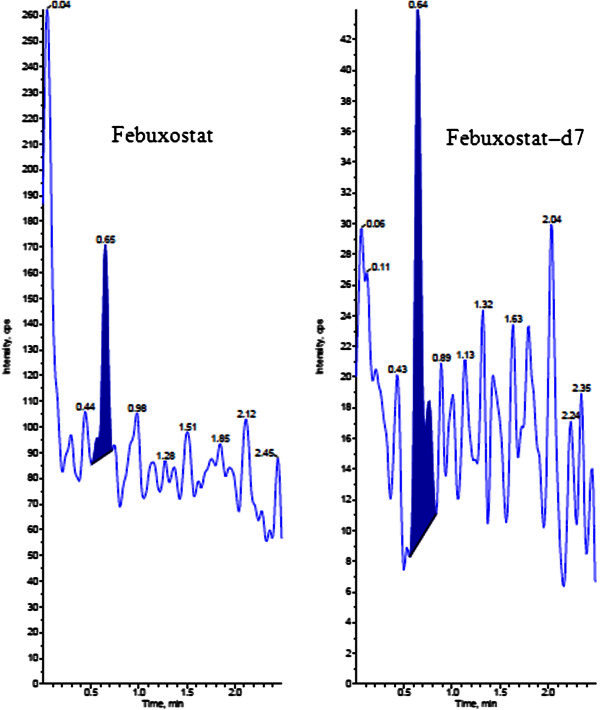
**Chromatogram of blank human plasma.**

**Figure 4:**
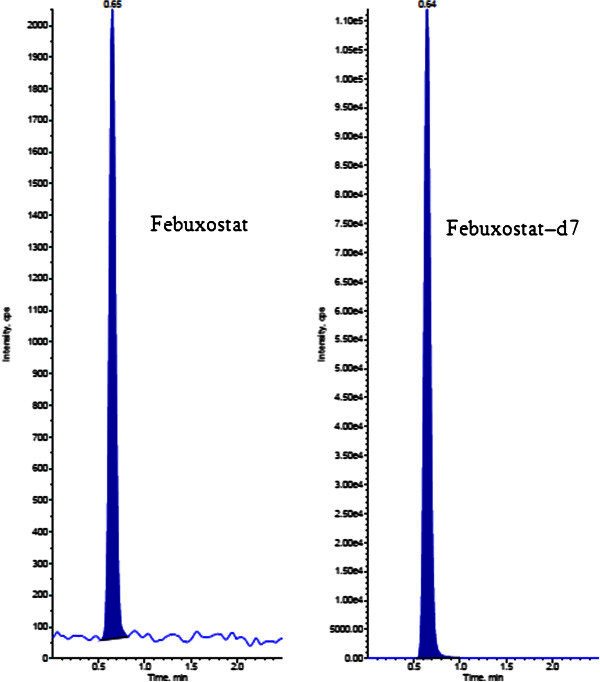
**LLOQ Chromatograms of Febuxostat and Febuxostat-d7.**

The analysis of FB and FBD7 using MRM (Multiple reaction monitoring) function was highly selective with no interfering compounds (Figure 3).Chromatograms obtained from plasma spiked with FB (1.00 ng/mL) and FBD7 (1000.00 ng/mL) are shown in Figure 4.

### Limit of detection (LOD) and quantification (LOQ)

The limit of detection was used to determine the instrument detection levels for FB even at low concentrations. 5 μL of a 0.5 pg/mL solution was injected and estimated LOD was 2.5 fg with S/N values ≥ 3–5.

The limit of quantification for this method was proven as the lowest concentration of the calibration curve which was proven as 1.00 ng/mL.

### Matrix effect

Six lots of blank biological matrices were extracted each in triplicates and post spiked with the aqueous standard at the mid QC level, and compared with neat standards of same concentration in alternate injections. The overall precision of the matrix factor is 5.67 for Febuxostat. There was no ion- suppression and ion- Enhancement effect observed due to IS and analyte at respective retention time.

### Precision and accuracy

Calibration curves were plotted as the peak area ratio (FB/FBD7) versus (FB) concentration. Precision and Accuracy of Calibration curve standards, Quality control standards represented in Tables 1 and 2.

**Table 1:** **Calibration curve details**

Spiked plasma concentration (ng/mL)	Concentration measured(ng/mL) mean	SD	CV (%) (***n*** = 5)	Accuracy %
1.00	1.02	0.03	2.72	102.20
2.00	1.93	0.12	5.99	96.50
20.00	19.46	0.45	2.32	97.30
100.00	98.50	1.87	1.90	98.50
200.00	209.80	4.22	2.01	104.90
400.00	411.20	15.33	3.73	102.80
1200.00	1222.00	14.83	1.21	101.83
2400.00	2390.00	62.05	2.60	99.58
3600.00	3592.00	67.60	1.88	99.78
6000.00	5932.50	125.19	2.11	98.88
8000.00	7813.33	132.66	1.70	97.67

**Table 2:** **Precision and accuracy at four different concentrations**

Spiked plasma concen-tration (ng/mL)	Within-run			Between-run		
	Concentration measured	CV^a^(%)	Accuracy %	Concentration measured	CV.^a^(%)	Accuracy %
	(***n*** = 6)			(***n*** = 30)		
	(ng/mL)			(ng/mL)		
	(mean ± S.d.)			(mean ± S.D.)		
1.00	0.98 ± 0.04	3.88	98.43	1.03 ± 0.09	8.44	103.19
3.00	2.92 ± 0.11	3.68	97.33	3.02 ± 0.17	5.57	100.78
4000.00	3957.78 ± 104.45	2.64	98.94	4021.78 ± 117.78	2.93	100.54
4800.00	4754.29 ± 158.36	3.33	99.05	4814.48 ± 133.07	2.76	100.30

^a^(Standard deviation/mean concentration measured)x100.

### Recovery

The recovery following the sample preparation using Liquid-liquid extraction with Methyl tertiary butyle ether was calculated by comparing the peak area of FB in plasma samples with the peak area of solvent samples and was estimated at control levels of FB. The recovery of FB was determined at three different concentrations 3.0, 4000.0 and 4800.0 ng/mL were found as 80.05, 84.02 and 80.67% respectively. The overall average recovery of FB and FBD7 were found to be 81.59 and 89.28% respectively.

### Stability (Freeze - thaw, Auto sampler, Bench top, Long term)

Quantification of the FB in plasma subjected to three freeze-thaw cycles (−30°C to room temperature), Autosampler, Room temperature(Benchtop), Long term stability details were shown in Table 3.

**Table 3:** **Stability of the samples**

Spiked plasma concentration (ng/mL)	Room temperature stability		Processed sample stability		Long term stability		Freeze and thaw stability	
	24.5 h		71.5 h		55 days		Cycle 3 (48 h)	
	Concentration measured	CV.^a^(***n*** = 6) (%)	Concentration measured	CV^a^(***n*** = 6) (%)	Concentration measured	CV.^a^(***n*** = 6) (%)	Concentration measured	CV.^a^(***n*** = 6) (%)
	(***n*** = 6)		(***n*** = 6)		(***n*** = 6)		(***n*** = 6)	
	(ng/mL)		(ng/mL)		(ng/mL)		(ng/mL)	
	(mean ± S.D)		(mean ± S.D)		(mean ± S.D)		(mean ± S.D)	
3.00	3.09 ± 0.16	5.26	3.11 ± 0.12	4.00	3.03 ± 0.08	2.56	3.04 ± 0.16	5.11
4800.00	5034.29 ± 62.49	1.24	4800.00 ± 104.74	2.18	4800.80 ± 172.50	3.59	4923.81 ± 148.20	3.01

^a^ (Standard deviation/mean concentration measured)X100.

### Application to biological samples

**Figure 5:**
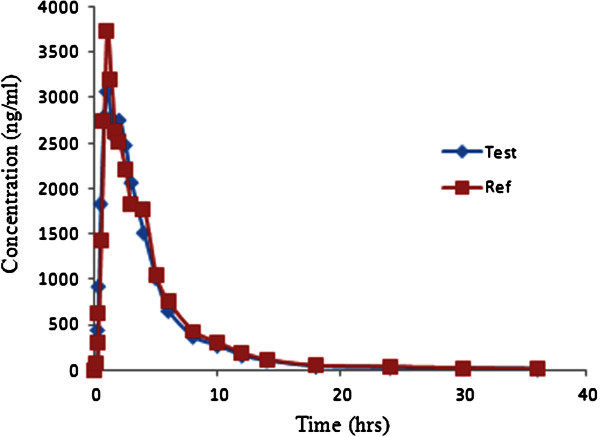
**Mean plasma concentrations of Test vs Reference after 80 mg dose(one 80 mg tablet) in 14 healthy volunteers.**

The above validated method was used in the determination of FB in plasma samples for establishing the bioequivalence of a single 80 mg dose (one 80 mg tablet) in 14 healthy volunteers. Typical plasma concentration versus time profiles is shown in Figure 5. All the plasma concentrations of FB were within the standard curve region and retained above the 10.0 ng/mL (LOQ) for the entire sampling period (Tables 4 and 5). The ANOVA results revealed that period, sequence and treatment had no statistically significant effects on C_max_, AUC_0-tlast_ and AUC_0-∞_. Since the sequence or carry-over effect was not significant, the ANOVA test was valid. The statistically significant subject within sequence effect on C_max_, AUC_0-tlast_ and AUC_0-∞_ were observed that are usually seen in small sample size study as in crossed over phase I and bioequivalence studies. Bioequivalence between the 80 mg immediate release tablet formulations of Febuxostat under fasting condition was demonstrated by the 90% CI of the geometric mean ratios of C_max_, AUC_0-tlast_ and AUC_0-∞_ lying within the acceptable criteria of 80-125%. The test and reference formulations had very similar t_1/2_ at approximately 6.5 h. Period, sequence and treatment had no significant effects on C_max_, AUC_0-tlast_ and AUC_0-∞_.

**Table 4:** **Mean pharmacokinetic parameters of febuxostat in 14 healthy volunteers after oral administration of 80 mg (1x80 mg) test and reference products**

Pharmacokinetic parameter	Febuxostat	
	Test	Reference
AUC_0-t_ (ng · h/ml)	13774.96	14367.34
Cmax (ng/mL)	3065.46	3726.09
AUC_0-∞_ (ng · h/ml)	13872.24	14469.30
Kel (h_1)	0.09983	0.10996
Tmax (h)	1	1

AUC_0—∞:_ area under the curve extrapolated to infinity;

AUC_0—t_: area under the curve up to the last sampling time;

Cmax: the maximum plasma concentration;

Tmax: the time to reach peak concentration;

Kel: the apparent elimination rate constant.

**Table 5:** **Test/Reference values for Log-transformed pharmacokinetic parameters of febuxostat after administration of 80 mg (1x80 mg) of test and reference products in 14 healthy male volunteers**

Pharmacokinetic parameters	Cmax	AUC_0-t_	AUC_0 - ∞_
Test/Ref	82.27	95.88	95.87

## Conclusion

The method described in this manuscript has been developed and validated over the concentration range of 1.00 - 8000.00 ng/mL in human plasma. The selectivity, sensitivity, precision and accuracy obtained with this method make it suitable for the purpose of the present study. The simplicity of the method, and using rapid liquid–liquid extraction and sample turnover rate of 2.5 min per sample, make it an attractive procedure in high-throughput bioanalysis of Febuxostat. The validated method was successfully applied and demonstrated the bioequivalence of the 80-mg immediate release tablet formulation manufactured by APL Research center., India and the reference product Urolac® manufactured by Takeda Pharmaceuticals, America,INC(USA) by oral administration in 14 healthy human volunteers, and then can be concluded that the two formulations can be used interchangeably.
